# Round pneumonia in an elderly woman

**DOI:** 10.1002/ccr3.42

**Published:** 2013-12-26

**Authors:** Tashfeen Mahmood, Adeline J Jou-Tindou, Faisal A Khasawneh

**Affiliations:** 1 Department of Internal Medicine, Deaconess HospitalEvansville, Indiana; 2 Northwest Texas Hospital, Amarillo Hospitalist groupAmarillo, Texas; 3 Section of Pulmonary and Critical Care Medicine, Department of Internal Medicine, Texas Tech University Health Sciences CenterAmarillo, Texas

**Keywords:** Lung infections, round pneumonia.

## Abstract

**Key Clinical Message:**

We describe a case of pneumococcal round pneumonia in an elderly smoker and it demonstrates the role of inflammatory biomarkers and follow-up imaging in ruling out more ominous diagnoses.

## Case

**Figure 1:**
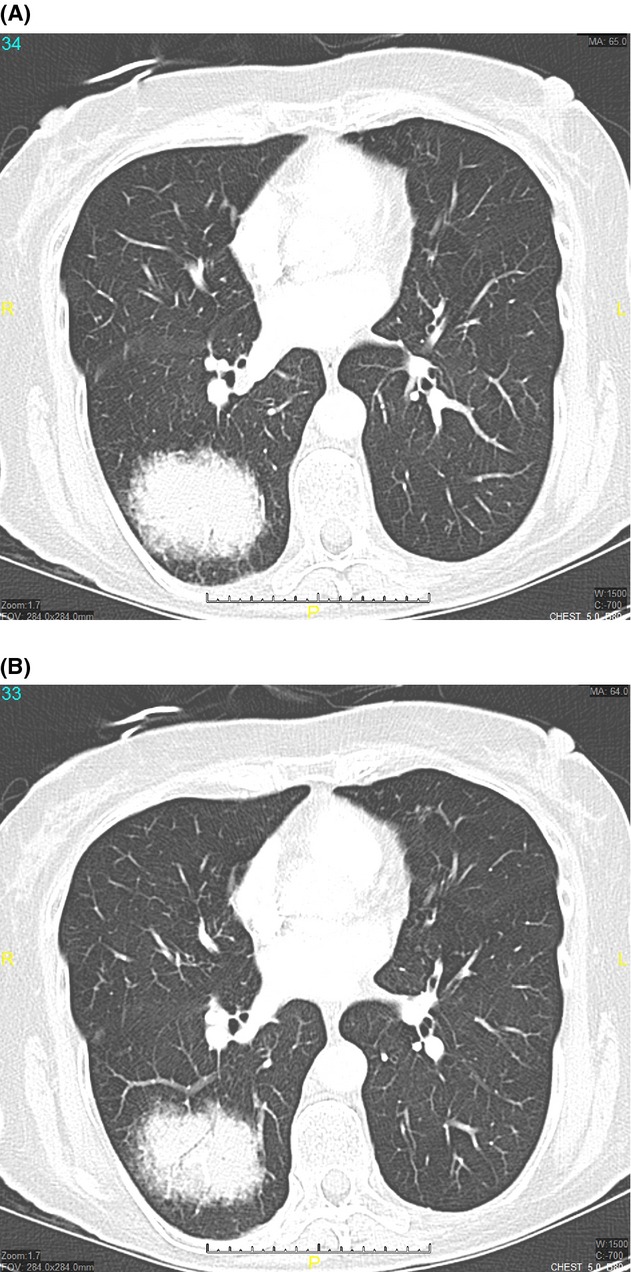
(A and B) Computed tomography (CT) scan cross sections showing round consolidation with air bronchogram.

A 74-year-old smoker female presented with dry cough and shortness of breath. Physical examination identified right lung base crackles. The patient's white blood cell counts were 11,700/*μ*L. Plain chest radiograph revealed right base infiltrate. A computed tomography (CT) scan of the chest is shown (Fig.1A and B).

What is the diagnosis?

Answer: Round pneumonia.

**Figure 2:**
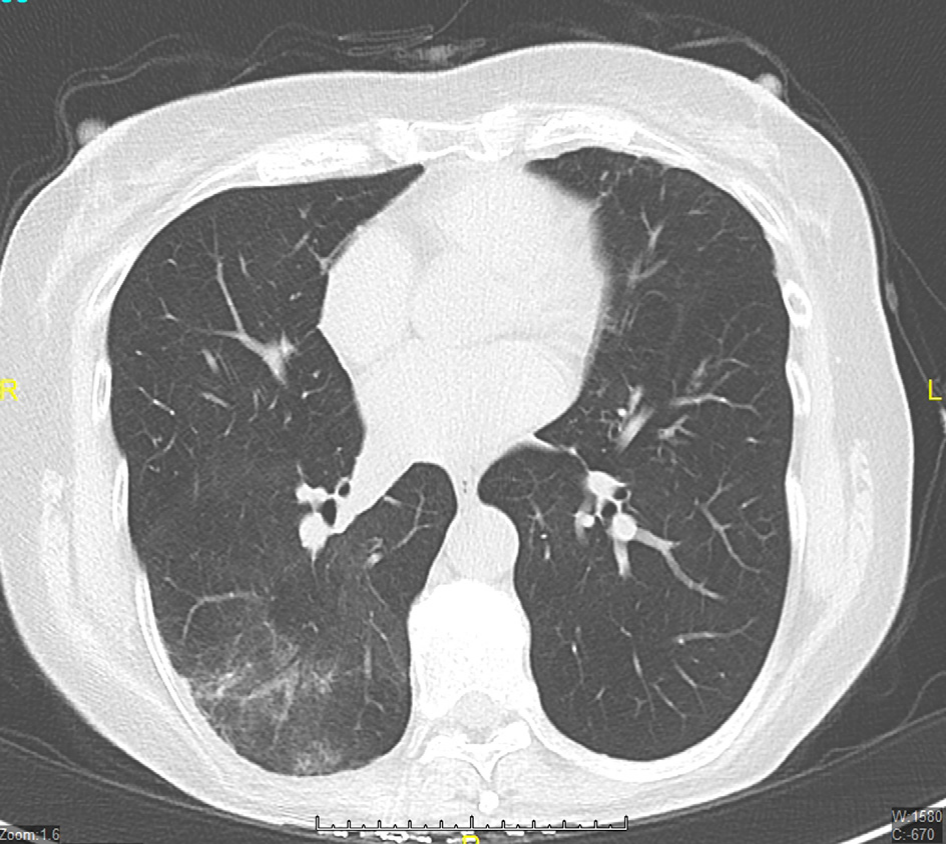
Follow-up CT scan cross section showing near-complete resolution of the consolidation. The test was carried out 8 weeks later.

Explanation:

The patient's blood cultures were negative, but serum procalcitonin level was elevated and urine pneumococcal antigen was positive. The patient's infection resolved with antibiotic therapy (Fig.2).

Round pneumonia is rarely seen in adults because of the development of collateral airways (pores of Kohn and canals of Lambert) by the age of eight [1]. It is a well-defined round consolidation that tends to occur in the upper segments of the lower lobes. Round pneumonia is mostly solitary (98%) and cavitation is not a common feature [2]. *Streptococcus pneumoniae* is the most common culprit in children and *Coxiella burnetii* and *Legionella micdadei* are commonly seen in adults [1]. Differential diagnoses include fungal and mycobacterial infections, round atelectasis, plural fibroma, bronchogenic cyst, pulmonary pseudotumor, and malignancy [1].
